# Health insurance fraud detection based on multi-channel heterogeneous graph structure learning

**DOI:** 10.1016/j.heliyon.2024.e30045

**Published:** 2024-04-24

**Authors:** Binsheng Hong, Ping Lu, Hang Xu, Jiangtao Lu, Kaibiao Lin, Fan Yang

**Affiliations:** a School of Computer and Information Engineering, Xiamen University of Technology, Xiamen, 361024, Fujian Province, China; b School of Economic and Management, Xiamen University of Technology, Xiamen, 361024, Fujian Province, China; c Zhongshan Hospital Xiamen University, Xiamen, Fujian Province, China; d Dept. of Automation, Xiamen University, Xiamen, Fujian Province, China

**Keywords:** Health insurance, Fraud detection, Heterogeneous graph neural networks, Graph convolutional network, Graph structure learning

## Abstract

Health insurance fraud is becoming more common and impacting the fairness and sustainability of the health insurance system. Traditional health insurance fraud detection primarily relies on recognizing established data patterns. However, with the ever-expanding and complex nature of health insurance data, it is difficult for these traditional methods to effectively capture evolving fraudulent activity and tactics and keep pace with the constant improvements and innovations of fraudsters. As a result, there is an urgent need for more accurate and flexible analytics to detect potential fraud. To address this, the Multi-channel Heterogeneous Graph Structured Learning-based health insurance fraud detection method (MHGSL) was proposed. MHGSL constructs a graph of health insurance data from various entities, such as patients, departments, and medicines, and employs graph structure learning to extract topological structure, features, and semantic information to construct multiple graphs that reflect the diversity and complexity of the data. We utilize deep learning methods such as heterogeneous graph neural networks and graph convolutional neural networks to combine multi-channel information transfer and feature fusion to detect anomalies in health insurance data. The results of extensive experiments on real health insurance data demonstrate that MHGSL achieves a high level of accuracy in detecting potential fraud, which is better than existing methods, and is able to quickly and accurately identify patients with fraudulent behaviors to avoid loss of health insurance funds. Experiments have shown that multi-channel heterogeneous graph structure learning in MHGSL can be very helpful for health insurance fraud detection. It provides a promising solution for detecting health insurance fraud and improving the fairness and sustainability of the health insurance system. Subsequent research on fraud detection methods can consider semantic information between patients and different types of entities.

## Highlights

• Extracting topology, feature information and semantic information from heterogeneous graphs of health insurance data to construct multi-views.
• By designing a meta-path common path sampling strategy to capture semantic information of patients connected by multiple meta-paths.
• Designing a multi-channel approach to learning the differences and commonalities between multiple views.

## 1 Introduction

In recent years, the global development of health insurance has not only ensured the health and well-being of people but also played a critical role in promoting social stability. Regrettably, the prevalence of health insurance fraud is a growing concern. According to the International Organization for Medicare Anti-Fraud, health insurance fraud leads to an annual loss of 260 billion dollars worldwide, equivalent to 6% of the total global health expenditure [1]. Data from the National Health Care Anti-Fraud Association suggests that between 3% to 10% of medical and health expenditures are wasted by fraud [2]. In China, the National Healthcare Security Administration inspected 708,000 designated medical institutions in 2021, handled 414,000 illegal institutions, and recovered 23.418 billion RMB of health insurance funds [3]. Moreover, a recent investigation by the National Healthcare Security Administration uncovered that Tongji Hospital had defrauded 23.34 million RMB in health insurance funds from January 2017 to September 2020 [4]. These fraudulent behaviors have caused a massive loss of funds, leading to a rapid increase in medical expenses globally. Despite the efforts made by the regulatory authorities, many fraudulent behaviors remain hidden in the complex health insurance data, making it challenging to identify potential fraudsters. Therefore, there is an urgent need to develop an efficient and accurate way to identify potentially fraudulent anomalous data.

Machine learning has demonstrated its potential for learning representations and delivering encouraging outcomes across a range of anomaly detection tasks [5], [6]. In the medical field, various approaches have been explored for health insurance fraud detection. Supervised learning-based methods such as the naive Bayes model [7] and random forest [8] have been used to identify fraudulent behavior. Unsupervised learning-based methods like the Bayesian hierarchical model [9] and the clustering method [10] have also been applied to detect potential fraud patterns. Additionally, some neural network-based approaches [11], multilayer perceptron-based neural networks [12], and knowledge graph-based models [13] have been widely used for health insurance fraud detection.

During the health insurance fraud detection process, traditional machine learning methods often struggle with sparse data and difficult feature extraction, making it challenging to leverage the heterogeneity of the data [14], [15]. However, converting raw health insurance data into graph data format and constructing heterogeneous graphs of health insurance data through the definition of nodes and edges can help uncover potential information. Health insurance participants, such as patients, department, time, and medicine, are represented as nodes in the graph, with rich information such as patient age and gender, department location, and medicine titles used as attributes between nodes for subsequent analysis. The nodes are then connected by edges, which can represent various interactions such as consultations, prescriptions, and reimbursements. This approach allows for identifying issues like over-prescription of medicines by certain patients by analyzing their relationship with medicines or over-reimbursement between specific hospitals by studying their interactions. Graph-based analytic methods can be a valuable tool for detecting and combating health insurance fraud by uncovering potential fraud networks and patterns through analyzing the interactions and influences among health participants.

In recent years, graph neural networks have garnered increasing attention in anomaly detection [16], [17], [18]. These methods represent the objects in the data as nodes, the relationships between them as edges, use graph neural networks to score anomalies appropriately, and identify data that may contain anomalous behavior. Heterogeneous graph neural networks (HGNNs) possess stronger expressive power than graph neural networks as they can effectively connect different types of nodes and edges, preserving graph structure and semantic information. Given the diverse characteristics of health insurance data, it can be effectively processed using a heterogeneous graph neural network. The use of heterogeneous graph methods to detect anomalies in health insurance data can help identify potential problems and anomalies, ensuring the quality and integrity of health insurance data and providing more reliable data support for decision-making in the field of health insurance.

**Figure 1:**
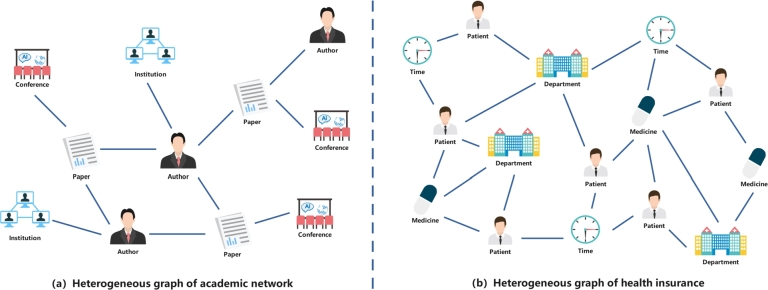
Example of a heterogeneous graph of academic network and health insurance data: (a) Heterogeneous graph of academic network; (b) Heterogeneous graph of health insurance data.

As shown in Fig. 1, both common academic networks and health insurance data heterogeneous graphs are heterogeneous in nature, but they are networks composed of different types of nodes and edges, and there are significant differences in the structures between them. The academic network heterogeneous graph is shown in Fig. 1(a), where nodes represent different types of entities, such as papers, authors, journals, etc., and edges represent the relationships between them, such as citations, collaborations, etc., with a relatively single data source. The heterogeneous graph of health insurance data is illustrated in Fig. 1(b). In this graphical representation, nodes correspond to various entities, including patients, departments, medicines, and times, while edges denote relationships such as visits, prescriptions, and more, resulting in a complex and diverse structure. Specifically, patients represent individuals seeking medical attention, departments signify the locations where these medical encounters occur, time nodes capture the temporal aspect of visits, and medicine nodes encapsulate medications prescribed by healthcare professionals. The heterogeneous nature of academic networks and health insurance data makes their characteristics in the application domain different. The purpose of analyzing academic heterogeneous graphs is usually for literature retrieval to study the relationships between literature and authors in academic fields in order to evaluate research results and explore the frontier directions and trends in academic fields. In contrast, analyzing health insurance heterogeneous graphs focuses on understanding entities and relationships in the health insurance domain and is used to study data relationships in the health insurance domain and uncover potential disease risks and treatment patterns for better health insurance decisions. As a new heterogeneous graph model with unique characteristics and application scenarios, the study of health insurance data heterogeneous graphs is important for both medical research and health insurance management.

Recent heterogeneous graph-based approaches to health insurance fraud detection have been limited by a number of challenges. These methods focus on identifying possible frauds by calculating the importance of nodes and using anomaly detection algorithms [19], [20]. However, the traditional heterogeneous graph approach requires processing large-scale health insurance data, which leads to a very high computational cost and slow processing speed. This makes it difficult to detect fraud in real-time and could result in missed fraudulent activities [21]. Moreover, conventional heterogeneous graph approaches necessitate the construction of intricate models, demanding substantial expertise and experience. This can be a challenge, especially in resource-limited settings where there may be a shortage of skilled personnel. Furthermore, these methods rely on manual extraction and selection of features, which are difficult to extract and select comprehensively and accurately because of the wide variety of features in health insurance data [22]. Therefore, there is a need for new methods and techniques that can overcome these challenges and improve the detection of anomalies in health insurance data.

Health insurance fraud detection is a complex task that necessitates the analysis of diverse data sources, such as patient medical histories and expenditure details, which often exhibit heterogeneity. Leveraging HGNNs offers a significant advantage in this context, as they are adept at managing and synthesizing varied data types. This capability is pivotal for enhancing the model's learning efficacy, particularly in the realm of health insurance fraud detection. Fraudulent activities can span across multiple data sources, and HGNNs facilitate a more holistic understanding of the relationships between these disparate pieces of information, thereby bolstering the detection process. Health insurance fraud is characterized by intricate interconnections among various stakeholders, including the dynamics between patients and hospital departments. HGNNs excel at deciphering these complex relationships by analyzing the nuanced interactions between entities through the graph's node and edge attributes. This analytical prowess is crucial for identifying health insurance fraud, given that fraudulent activities frequently entail collaborative efforts among multiple parties. By employing HGNNs, we can uncover the subtle synergistic patterns that often underlie such fraudulent schemes. Leveraging heterogeneous graph structure learning methodologies enables the seamless integration of such information, allowing for precise identification of the nuanced interconnections between multiple entities within the realm of health insurance data. Consequently, the implementation of these learning techniques in the detection of health insurance fraud significantly enhances the precision of fraud detection models, effectively tackling the multifaceted and complex challenges inherent to the health insurance sector.

In this paper, we present a method for health insurance fraud detection based on Multi-channel Heterogeneous Graph Structured Learning (MHGSL). Our approach is designed to extract rich features, structures, and semantic information from heterogeneous graphs, which can capture complex relationships between different types of entities, including multiple types of nodes and edges. Compared to previous heterogeneous graph methods, we analyze the entire heterogeneous graph, rather than focusing on individual entities or edges, and are able to can better understand the global relationships between entities in the health insurance data. This allows us to detect anomalies in the overall data and identify possible holistic problems and anomalies, thus improving the accuracy and efficiency of the analysis.

In our proposed method, the first step is to construct a heterogeneous graph using health insurance data. To capture deep structural information about nodes in feature space, we calculate the cosine similarity between n-nodes and use the K-nearest neighbor method (KNN) [23] to select the first *k* similar neighbor nodes for each sample node to set edges. This generates a new adjacency matrix, which we combine with the features to create a KNN feature graph. We also aim to preserve the relationships between nodes in the original graph and obtain structural information on the health insurance heterogeneous graph layer, so we extract the topological relations between sample nodes to build a node topology graph. In addition, we aim to capture high-order similarity and hidden semantic information between nodes in the heterogeneous graph of health insurance data, so we sample and extract different types of meta-paths. However, meta-paths alone cannot express dependencies on complex nodes. Therefore, we use common path sampling for different types of meta-paths to capture complex semantic relations, generate semantic adjacency matrices, and construct semantic graphs. This approach enables us to capture complex relationships and better understand the semantic information between nodes in the heterogeneous graph of health insurance data.

Previous methods for learning graph structures only considered the embedding of features learned in the feature space or topological space [24]. However, in reality, the different spaces are not completely independent, and the anomaly detection task may be related to the information in each space, partially or completely. To address this, we propose a multi-channel approach to obtain embeddings of different types of graph structures propagated in topological space, feature space, and semantic space. Our method includes two types of Graph convolutional network (GCN) [25] channels: a single GCN channel for learning the information of a single graph in the corresponding space, and a common GCN channel with shared parameters for learning the common information of different types of graphs in the same space. This enables efficient learning of the differences and commonalities between graphs of the same type in different spaces and between graphs of different types in the same space during propagation.

Finally, we introduce an attention mechanism that enables the adaptive weighting of embeddings learned in different spaces. This mechanism allows the model to focus on the most relevant information for the downstream task and effectively fuse all embedding information. Specifically, we use a fully connected layer to graph the embeddings learned from different channels into a common space and then apply an activation function to the resulting vector to obtain the attention weights. These weights are then used to linearly combine the embeddings, producing a final fused representation for each node in the heterogeneous graph. This adaptive fusion of information allows the model to effectively utilize the complementary information from different spaces, leading to improved performance in downstream tasks such as health insurance fraud detection.

There are several major contributions of this paper, which can be summarized as follows:

(1) This paper proposes a novel health insurance fraud detection method, named MHGSL. It extracts network topology, node features and semantic information in heterogeneous health graphs and constructs various types of graph structures.
(2) In the semantic graph constructing process, present study perform common path sampling on meta-paths to efficiently capture higher-order similarities between nodes and obtain semantic information between patients with multiple meta-path connections.
(3) To learn the differences of the same type of graph structures in different spaces, as well as the commonalities in different types of graph structures in the same space, a multi-channel approach was designed.
(4) To proof the excellent performance of MHGSL model, extensive experiments on real health insurance datasets were executed.

## 2 Related work

The aim of health insurance fraud detection is to identify abnormal samples from large and complex health insurance datasets, and numerous fraud detection methods have been proposed. These methods can be broadly categorized into five groups: rule-based methods, supervised learning-based methods, unsupervised methods, neural network-based methods, and graph network-based methods.

Rule-based methods [26], [27] leverage domain knowledge to identify behavioral patterns and create rules for detecting those patterns and filtering abnormal data. For example, Sadiq et al. [28] proposed a bump search method using the Patient Rule Induction Method (PRIM) to detect abnormal peaks by identifying higher-order patterns. Kirlidog et al. [29] calculated the probability of fraudulent behavior for each data point, followed by in-depth investigations. While rule-based methods are effective, they depend on predefined rules to identify all abnormal signals, and hidden abnormalities in complex health insurance data may go unnoticed. Supervised learning methods [30], [31] focus on learning features from a limited number of anomalous samples, heavily training the model on the dataset for fraud prediction. For instance, Severino and Peng [32] explored ensemble methods using random forest, gradient boosting, and deep learning networks, demonstrating superior performance in estimating feature impact on global classification. Kannagi et al. [33] introduced a PR-KNN network utilizing progressive search and non-parametric techniques to optimize solutions and reduce fraud. Nevertheless, these supervised approaches may lack sensitivity to outliers and overlook the computational and spatial complexities associated with large datasets.

Unsupervised learning methods [34], [35] prove effective in capturing behavioral features of abnormal data and isolating outliers, making them well-suited for health insurance fraud detection. For instance, Ekin et al. [9] proposed an unsupervised Bayesian hierarchical method for assessing health insurance fraud, focusing on outlier detection and similarity evaluation. However, its high computational cost restricts its applicability to low-dimensional data, limiting the exploration of higher-dimensional information. Additionally, these methods may necessitate obvious density differences in the detected data, posing a limitation. Zhang et al. [36] proposed an enhanced density-based local outlier detection algorithm addressing edge misjudgment and outlier issues arising from clusters with varying densities. Nevertheless, it exhibits computational complexity and may not be suitable for large datasets.

Neural network-based approaches [37], [38] aim to detect fraudulent behavior by adjusting interconnections among numerous internal nodes, facilitating distributed parallel information processing. Pioneered by He et al. [39] the application of neural networks in health insurance fraud detection involves classifying data samples into normal and abnormal using a multilayer perceptron. They also proposed a fraud detection application combining genetic and nearest neighbor algorithms, which determines optimal weights for classification features and employs these weights for identifying nearest neighbor data [40]. However, these methods are susceptible to local minima, gradient dispersion, and inefficient utilization of network feedback information. In a different approach, Cao et al. [41] utilized Principal Component Analysis (PCA) to reduce feature data dimensionality and established a Self-Organizing Feature Map (SOFM) neural network for health insurance fraud detection. While achieving more accurate results when the prediction is unknown, this method faces challenges due to the complex learning process of building a neural network and a prolonged clustering time requirement.

Graph network-based methods [42], [43], [44] for anomaly detection leverage the power of graph neural networks to model graph-structured data efficiently. Wang et al. [16] introduced a classification framework that effectively detected anomalies by mapping training nodes into a vector space using graph neural networks. However, this method is constrained to static nodes. Xie et al. [17] enhanced anomaly detection in time series data by converting high-dimensional time series into graph data, improving detection performance. Nevertheless, there is room for improvement in both the model's generalization ability and the data transformation process. Qiu et al. [18] introduced an outlier detection algorithm employing graph convolution and attention to tackle feature ambiguity and improve accuracy. While effective in numerous outlier detection tasks, its suitability for more complex scenarios may be limited. Chen et al. [45] suggested constructing a patient network based on behavioral patterns, transforming heterogeneous information into homogeneous information. However, the resulting patient network might lack complexity in behavioral patterns, limiting its richness.

Recent research in heterogeneous graph representation-based learning has concentrated on preserving both graph structure and feature information. Wang et al. [24] investigated the fusion mechanism of topology and node features in graph convolution networks, suggesting adaptive learning of the most relevant information from both aspects. Zhao et al. [46] proposed a model for joint learning of heterogeneous graph structures and Graph Neural Networks (GNN) parameters to learn an appropriate graph structure for heterogeneous graph neural networks. Wang et al. [47] presented a multi-graph convolution clustering network utilizing single-graph attention and multi-graph attention to emphasize node and graph importance, respectively. Gao et al. [48] developed a method for extracting topological features through clustering coefficients, enhancing the effective utilization of topological and node features in the graph convolution process. Heterogeneous directed graphs are also widely used in a variety of complex networks, which can more comprehensively capture the relationships between different node types and directed edges in the graph data, and thus more accurately characterize the structure of complex systems. Liu et al. [49] proposed a method of always semantic proximity search to represent the proximity between two nodes by learning the embedding of a directed acyclic graph to address the limitations of traditional representations in expressing complex network connections. Liang et al. [50] concentrate on learning causal relationships within attribute-heterogeneous networks by focusing on directed acyclic graphs. They use contrast learning in conjunction with a pre-existing network structure to explore potential relationships between nodes and update node representations. Dealing with heterogeneous directed graphs involves navigating the complexity of their relational representation. Choosing an appropriate model and tuning it for a specific task are crucial considerations in this context. In practical health care fraud detection, challenges arise in modeling complex relationships within directed graphs involving multiple node and edge types.

Recently proposed graph contrast learning methods leverage the contrast learning principle to acquire node representations in heterogeneous graphs [51], [52]. Chen et al. [53] applied a contrast learning approach to enhance heterogeneous graph learning in recommender systems, aiming to improve performance. However, challenges related to computation and data dependency exist. Cai et al. [54] investigated the use of heterogeneous graph contrast learning in video recommendation, recognizing its potential to improve the recommendation experience while emphasizing challenges such as data sparsity and model complexity. Wang et al. [55] propose a novel self-supervised heterogeneous graph neural network that uses self-supervised contrast learning to solve the node embedding learning problem in heterogeneous information networks across views. Jiang et al. [56] proposed a pre-trained graph neural network for heterogeneous graph comparison to solve the label scarcity problem faced by graph neural networks when dealing with heterogeneous graphs. Chen et al. [57] proposed a new heterogeneous graph comparison learning that enhances representation learning in heterogeneous relation learning by combining a heterogeneous graph neural network and a cross-view comparison learning paradigm. While heterogeneous graph-based contrast learning efficiently models multiple node and edge types, its application to health insurance data encounters challenges due to diverse semantic relationships and a lack of fine-grained control over labels. In response, our study investigates complex health insurance heterogeneous graphs, exploring topological, node, and semantic features and examining combinations of different feature types.

In the current research field, most of the approaches focus on single type of information processing, or when dealing with multiple types of information, they often lack in-depth mining of complex relationships among different information. Existing literature usually uses linear or simple nonlinear models to integrate different types of data, and these approaches have obvious limitations in capturing complex interactions and higher-order similarities among data. However, real-world information is often multidimensional and interconnected, which requires a more granular approach to process and integrate this information. The research in this paper proposes an innovative approach to processing and integrating different types of information by constructing different types of graphs and utilizing the complex structure of graph networks. Our method simultaneously learns the shared features among different types of information, thereby enhancing the model's ability to integrate diverse information types. To extract semantic information, our method samples various types of meta-paths and performs common path sampling for each type. This generates multiple meta-paths that connect sample nodes, creating a semantic graph that captures high-order similarities between nodes.

## 3 Preliminary

This study aims to construct a heterogeneous graph of health insurance data, and analyze the graph to efficiently detect and identify patient samples in which there are abnormalities. Below we introduce some important terms related to heterogeneous graphs and some basic concepts and examples that will be used in this article.

**Heterogeneous Graph**

We denote the heterogeneous graph as G(V,E,X), where *V* represents the set of nodes, *E* represents the set of links, and *X* represents the set of node types attribute information [58]. Generally, nodes are randomly assigned as $v_{1},v_{2},v_{3},\ldots ,v_{n}$, where |V|=N. Here, *N* stands for the number of nodes, V→U signifies the mapping function for node types, while E→R denotes the mapping function for edge types. *U* and *R* represent the sets of predefined object types and link types, respectively, and there is |U|+|R|>2. To identify abnormal patients within the heterogeneous graph, the patient node is designated as the target node, while other nodes serve as neighbor nodes. The transformation of the heterogeneous graph G(V,E,X) into G(A,X) involves creating an adjacency matrix $A=[A_{ij}]$ for the heterogeneous graph *G*. The adjacency matrix $A=[A_{ij}]$ is a 0-1 matrix composed of *N* nodes, where $A_{ij}=1$ indicates adjacency between node $v_{i}$ and node $v_{j}$, and $A_{ij}=0$ signifies the absence of adjacency between node $v_{i}$ and node $v_{j}$.

**Figure 2:**
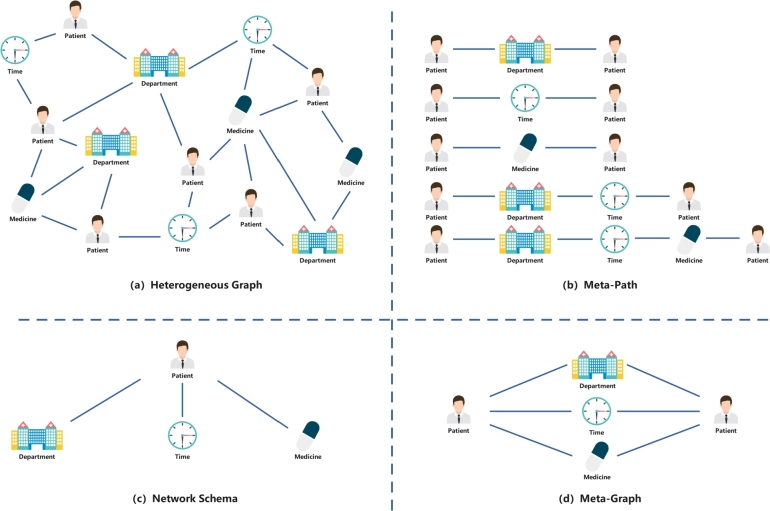
Examples of (a) Heterogeneous Graph; (b) Meta-Path; (c) Network Schema; and (d) Meta-Graph in health insurance dataset.

Example 1 As shown in Fig. 2 (a), we construct a heterogeneous graph example model for the health insurance dataset, which includes four types of nodes (patient, department, time, medicine) and six types of edges (patient-department, patient-time, patient-medicine, department-time, department-medicine, time-medicine).

**Heterogeneous Graph Embedding**

Given a heterogeneous graph G(V,E,X), map the nodes into a *D*-dimensional vector space via the function $V\to R^{D}$, and learn a representation that captures the structural and semantic information between nodes, where D≪|V| [59]. In simple terms, the goal of a heterogeneous graph embedding is to learn a function that graphs nodes in a heterogeneous graph into a low-dimensional vector space. While learning representations in low-dimensional spaces, the structure and semantics of heterogeneous graphs are preserved and applied to downstream tasks.

**Meta-Path**

A meta-path *P* is generally defined as a path $P=A_{1}\overset{R_{1}}{\to }A_{2}\overset{R_{2}}{\to }A_{3}\overset{R_{3}}{\to }\ldots \overset{R_{l}}{\to }A_{l+1}$ of the following form, which can also be abbreviated as $A_{1}A_{2}A_{3}\ldots A_{l+1}$ [60]. Use $R=R_{1}\circ R_{2}\circ R_{3}\circ \ldots \circ R_{l}$ to describe the compound relationship between node types $A_{1}$ and $A_{l+1}$, where ∘ represents the compound operator on the relationship.

Example 2 As shown in Fig. 2 (b), the meta-path instance we extracted from the heterogeneous graph model constructed from the health insurance dataset. The types of meta-paths involved are: patient-department-patient, patient-time-patient, patient-medicine-patient, patient-department-time-patient, patient-department-time-medicine-patient.

**Network Schema**

We denote the network schema as *S*, which is an undirected graph defined on node types and link edges, elucidating the relationships between various node types [61]. The network schema effectively transforms heterogeneous graphs into semi-structured networks.

Example 3 An example of a network schema is shown in Fig. 2 (c), a network schema consisting of patients, departments, times, and medicines. This semi-structured network represents a particular patient who visits a hospital department at a specific point in time to get a prescription and obtain medication.

**Meta-Graph**

We define the meta-graph [62] as $G_{\tau }=(V_{\tau },E_{\tau })$, where $V_{\tau }$ is the node audit and $E_{\tau }$ is the set of edges. A meta-graph $G_{\tau }$ can be viewed as a directed graph consisting of multiple meta-paths with common nodes.

Example 4 An example of a pattern is shown in Fig. 2 (d), a meta-graph consisting of three meta-paths: patient-department-patient, patient-time-patient, and patient-medicine-patient.

## 4 Methods

**Figure 3:**
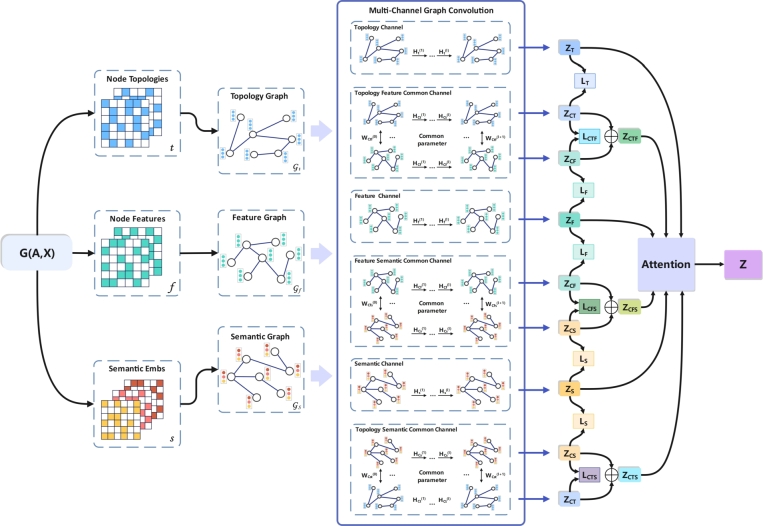
The overall framework of the MHGSL model.

We propose an innovative model called MHGSL for detecting health insurance fraud, which utilizes a multi-channel heterogeneous graph structure. Fig. 3 provides an overview of the entire framework.

### 4.1 Feature graph

In order to capture the deep structure information of node features in addition to the transformed heterogeneous graph *G*, we construct a KNN graph $G_{f}=(A_{f},X)$ based on the similarity between node features, where $A_{f}$ is the adjacency matrix of the KNN graph. To be more specific, we use the vector space-based cosine similarity method to calculate the similarity matrix *S*. The cosine similarity method measures the similarity between samples by evaluating the cosine value of the angle between the feature vectors $x_{i}$ and $x_{j}$ of node *i* and *j*. The cosine similarity is calculated as shown in Equation (1) below.

(1) $$S_{ij}=\frac{x_{i}\cdot x_{j}}{\left|x_{i}|\cdot |x_{j}\right|}$$

After calculating the similarity matrix *S*, we select the top *k* similar nodes for each node as the neighborhood of the node, thus obtaining the similarity matrix $A_{f}$ of KNN. Finally, the similarity matrix $A_{f}$ and feature *X* are combined into a feature graph $G_{f}=(A_{f},X)$.

### 4.2 Semantic graph

**Figure 4:**
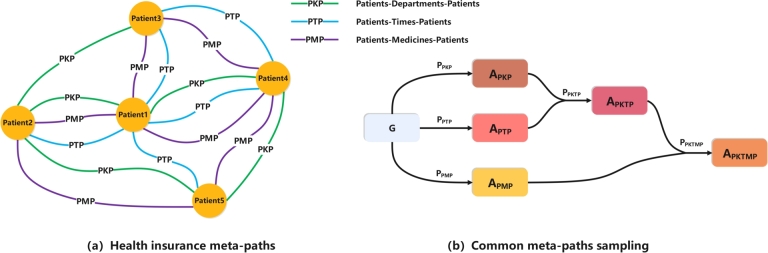
Meta-paths sampled in the health insurance heterogeneous graph and common path sampling process for meta-paths.

As shown in Fig. 4(a), in order to capture the hidden semantic information in the health insurance heterogeneous graph G, we sample different types of meta-paths $P_{PKP}$, $P_{PTP}$, $P_{PMP}$ from the health insurance heterogeneous graph. The meta-path $P_{PKP}$ is represented as shown in Equation (2) below.

(2) $$P_{PKP}=A_{P}\overset{R_{PK}}{\to }A_{K}\overset{R_{KP}}{\to }A_{P}=(A_{P},A_{K},A_{P})$$

These meta-paths are specific sequences of entity types and relationships that capture higher-order connectivity patterns between different types of entities in the graph. For example, the meta-path $P_{PKP}$ captures a pattern in which two different patients (denoted as *P*) are connected through a shared department (denoted as *K*) in the same hospital. This type of higher-order connectivity pattern can provide valuable insights into the relationships and interactions between entities in a graph, and can be used for a variety of applications such as recommendation systems, predictive modeling, and anomaly detection.

Meta-paths are useful in describing semantic relationships between nodes in a heterogeneous graph, but they may not be sufficient to capture dependencies between complex nodes. To overcome this limitation, we propose a method for sampling common paths for different types of meta-paths, which enables us to capture the complex semantic relationships within the health insurance network. Fig. 4(b) illustrates our approach for capturing complex semantic relationships in the health insurance network. We first sample the common path of the two meta-paths, $P_{PKP}$ and $P_{PTP}$, to obtain the meta-graph $P_{PKTP}=(P_{PKP},P_{PTP})$ that contains the semantic information of both meta-paths. Then the meta-path $P_{PMP}$ and the meta-graph $P_{PKTP}$ containing the semantic information of the two meta-paths are used for common path sampling. Obtain the meta-graph $P_{PKTMP}=(P_{PKP},P_{PTP},P_{PMP})$ that connects three different types of meta-paths simultaneously between patient nodes, and generate the corresponding multi-path semantic adjacency matrix $A_{PKTMP}$. Finally, we combine the semantic adjacency matrix $A_{s}=A_{PKTMP}$ connected by multiple meta-paths and the feature *X* into a semantic graph $G_{s}=(A_{s},X)$.

The construction of common meta-path semantic graphs is specialized for medical scenarios, where semantic graphs between patients are constructed by meta-path sampling and common path sampling, and deep semantic information is provided by mining the same behavioral patterns. Patients with fraudulent behaviors are usually similar in several aspects. Constructing a common meta-path semantic graph helps to reveal these similarities, which in turn provides more comprehensive and accurate information to help identify potential fraudulent behaviors. Common path sampling based on the aforementioned meta-paths is used to obtain deep semantic information between patients, aiming to mine the same behavioral patterns between different patients in this way. This customized meta-path sampling approach enables our model to reveal more accurately the relationships and behavioral patterns between patients in the health insurance domain.

### 4.3 Specific graph convolution

**Figure 5:**
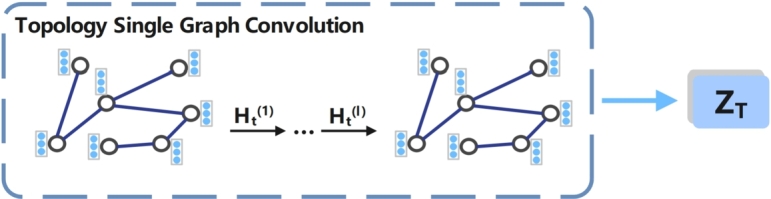
Learning node embedding processes in topological spaces.

In this section, we describe how we learn embeddings for specific node information in a designated space. To achieve this, we input the topology graph $G_{t}=(A_{t},X)$, the feature graph $G_{f}=(A_{f},X)$ and the semantic graph $G_{s}=(A_{s},X)$ into a specific graph convolution to learn embeddings. The process of learning node embeddings $Z_{T}$ in the topological space is illustrated in Fig. 5. We input the topology graph $G_{t}$ and feature *X* into topological space, and connect the two-layer GCN to learn the embedding of the topology graph in topological space. The embedding of the output of the *l*-th layer is $H_{t}^{\left(l\right)}$, which is calculated as shown in Equation (3) below.

(3) $$H_{t}^{\left(l\right)}=RELU({\overset{˜}{D_{t}}}^{-\frac{1}{2}}\overset{˜}{A_{t}}{\overset{˜}{D_{t}}}^{-\frac{1}{2}}H_{t}^{\left(l-1\right)}W_{t}^{\left(l\right)})$$

Among them, ${\overset{˜}{D_{t}}}^{-\frac{1}{2}}\overset{˜}{A_{t}}{\overset{˜}{D_{t}}}^{-\frac{1}{2}}$ is a quantity related to the network topology, and $H_{t}^{\left(0\right)}=X$ represents the characteristics of the initial node, $H_{t}^{\left(l-1\right)}$ is the feature of the (l−1)th layer node, $W_{t}^{\left(l\right)}$ is the weight matrix of the graph network in the *l*-th layer of the GCN, ReLu is the nonlinear activation function in the artificial neural network.

We denote the output embedding $H_{t}^{\left(l\right)}$ of the last layer as $Z_{T}$, $Z_{T}=H_{t}^{\left(l\right)}$ is the topology graph $G_{t}=(A_{t},X)$ learns to have specific topological information in a specific topological space node embedding. Similarly, input the feature graph $G_{f}=(A_{f},X)$ into the feature space through the above calculation method to learn the node embedding with specific feature information $Z_{F}=H_{f}^{\left(l\right)}$. Input the semantic graph $G_{s}=(A_{s},X)$ into the semantic space to learn node embeddings with specific semantic information $Z_{S}=H_{s}^{\left(l\right)}$. $H_{f}^{\left(l\right)}$ and $H_{s}^{\left(l\right)}$ are calculated as shown in Equation (4) and (5) below.

(4) $$H_{f}^{\left(l\right)}=RELU({\overset{˜}{D_{f}}}^{-\frac{1}{2}}\overset{˜}{A_{f}}{\overset{˜}{D_{f}}}^{-\frac{1}{2}}H_{f}^{\left(l-1\right)}W_{f}^{\left(l\right)})$$

(5) $$H_{s}^{\left(l\right)}=RELU({\overset{˜}{D_{s}}}^{-\frac{1}{2}}\overset{˜}{A_{s}}{\overset{˜}{D_{s}}}^{-\frac{1}{2}}H_{s}^{\left(l-1\right)}W_{s}^{\left(l\right)})$$

### 4.4 Shared parameter graph convolution

In fact, there may be many similarities among the embeddings learned in topological, feature, and semantic spaces, and downstream tasks may rely on common information in these spaces. Therefore, it is important to not only extract the specific embeddings learned by nodes in different types of spaces but also to identify and capture the common information shared between different types of spaces. In this study, we aim to learn features that are shared across different spaces by utilizing shared parameter graph convolutions. By sharing parameters, the graph convolutional layers can capture common patterns and information across the different types of graphs. This allows us to learn features that are shared between the different types of spaces and can be used for downstream tasks that require information from multiple spaces.

**Figure 6:**
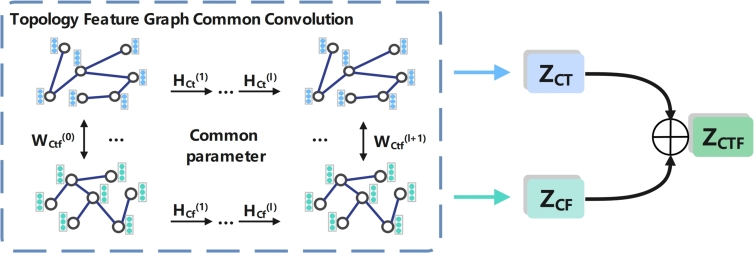
Shared parameter learning node topology and feature embedding process.

As illustrated in Fig. 6, we input the topology graph $G_{t}=(A_{t},X)$ into the shared parameters GCN module to learn the node embedding, and denote the output embedding $H_{ct}^{\left(l\right)}$ of the last layer as $Z_{CT}$. $H_{ct}^{\left(l\right)}$ is calculated as shown in Equation (6) below.

(6) $$H_{ct}^{\left(l\right)}=RELU({\overset{˜}{D_{t}}}^{-\frac{1}{2}}\overset{˜}{A_{t}}{\overset{˜}{D_{t}}}^{-\frac{1}{2}}H_{ct}^{\left(l-1\right)}W_{ct}^{\left(l\right)})$$

Among them, $H_{ct}^{\left(0\right)}=X$ represents the feature of the initial node in the topological space, $H_{ct}^{\left(l-1\right)}$ is the feature of the (l−1)th layer node, $W_{ctf}^{\left(l\right)}$ is the graph network in the shared weight matrix for the *l*-th layer in a common GCN module.

While inputting the topology graph $G_{t}=(A_{t},X)$ into the common GCN module to learn the node embedding, also input the feature graph $G_{f}=(A_{f},X)$ into the same common GCN module to learn the node embedding. In the process of learning embedding in GCN, the weight matrix of the *l*-th layer and the topology graph are shared in the common GCN module. The shared weight matrix is $W_{ctf}^{\left(l\right)}$, and the output embedding $H_{cf}^{\left(l\right)}$ of the last layer is denoted by $Z_{CF}$. The procedure for calculating $H_{cf}^{\left(l\right)}$ is shown in Equation (7) below.

(7) $$H_{cf}^{\left(l\right)}=RELU({\overset{˜}{D_{f}}}^{-\frac{1}{2}}\overset{˜}{A_{f}}{\overset{˜}{D_{f}}}^{-\frac{1}{2}}H_{cf}^{\left(l-1\right)}W_{ctf}^{\left(l\right)})$$

Among them, $H_{cf}^{\left(0\right)}=X$ represents the feature of the initial node in the feature space, and $H_{cf}^{\left(l-1\right)}=X$ is the feature of the (l−1)th layer node.

In the GCN module with shared parameters, the topology graph $G_{t}$ and the feature graph $G_{f}=(A_{f},X)$ are input, and two different embeddings $Z_{CT}$ and $Z_{CF}$ are obtained. We average the obtained two embeddings $Z_{CT}$ and $Z_{CF}$, and finally get the embedding $Z_{CTF}$ learned in this GCN module. $Z_{CTF}$ is calculated as shown in Equation (8) below.

(8) $$Z_{CTF}=Mean(Z_{CT},Z_{CF})$$

Similarly, we use the same method as above to input the topology graph $G_{t}$ and the semantic graph $G_{s}$ into the common GCN module to learn the embedding $Z_{CT}$ and $Z_{CS}$ by sharing the weight matrix, and average $Z_{CT}$ and $Z_{CS}$ these two embeddings to get the final embedding $Z_{CTS}$. At the same time, the feature graph $G_{f}$ and the semantic graph $G_{s}$ are input into the common GCN module, and the embeddings $Z_{CF}$ and $Z_{CS}$ of two different spaces are learned by sharing the weight matrix. Finally, average $Z_{CF}$ and $Z_{CS}$ these two embeddings to get the final embedding $Z_{CFS}$.

### 4.5 Multi-graph attention mechanism

Embeddings are learned by feeding topology graph, feature graph and semantic graph into a specific graph convolution module and a shared parameter graph convolution module. Now we have three specific embeddings $Z_{T}$, $Z_{F}$ and $Z_{S}$ obtained in a specific GCN graph convolution module, and three other embeddings $Z_{CTF}$, $Z_{CTS}$ and $Z_{CFS}$ obtained in a shared parameter graph convolution module.

To account for potential correlations between the learned embeddings of different graphs, we incorporate an adaptive weighted attention mechanism, denoted as $a_{Attention}$, to learn the relative importance of these embeddings. $a_{Attention}$ is calculated as shown in Equation (9) below.

(9) $$a_{Attention}=(a_{T},a_{F},a_{S},a_{CTF},a_{CTS},a_{CFS})=att(Z_{T},Z_{F},Z_{S},Z_{CTF},Z_{CTS},Z_{CFS})$$

Among them, the embeddings learned in different spaces and the corresponding attention weights are shown in Table 1. In order to fuse the information between multiple graphs, we combine the six embeddings and the corresponding attention weights to synthesize the final embedding *Z*. *Z* is calculated as shown in Equation (10), (11) and (12) below.

(10) $$Z_{dep}=a_{T}\cdot Z_{T}+a_{F}\cdot Z_{F}+a_{S}\cdot Z_{S}$$

(11) $$Z_{com}=a_{CTF}\cdot Z_{CTF}+a_{CTS}\cdot Z_{CTS}+a_{CFS}\cdot Z_{CFS}$$

(12) $$Z=Z_{dep}+Z_{com}$$

**Table 1:** Embeddings learned in different spaces and assigned attention weights.

Embeddings	Attention Weight
*Z*_*T*_	*a*_*T*_
*Z*_*F*_	*a*_*F*_
*Z*_*S*_	*a*_*S*_
*Z*_*CTF*_	*a*_*CTF*_
*Z*_*CTS*_	*a*_*CTS*_
*Z*_*CFS*_	*a*_*CFS*_

### 4.6 Loss function

In order to better complete the classification task of abnormal nodes, we define three loss functions: cross-entropy loss $L_{cross}$, difference loss $L_{similar}$ and consistency loss $L_{common}$ to constrain our model. First, we add a linear layer after embedding *Z* to reduce the dimension to the size of the node category, and the embedding *Z* is computed as shown in Equation (13) below.

(13) $$\overset{¯}{Y}=softmax(ZW)$$

$W\in R^{d\times c}$ is the weight matrix, and *c* is the number of categories. We assume that the training set is $V_{L}$, for each data sample node $v\in V_{L}$, the label of the predicted sample is ${\overset{¯}{Y}}_{l}$, the label of the actual sample is $Y_{l}$. The cross-entropy loss function can be expressed as shown in Equation (14) below.

(14) $$L_{cross}=-\sum_{v\in V_{L}}\sum_{i=1}^{C}Y_{l}[i]log{\overset{¯}{Y}}_{l}[i]$$

Second, the embeddings learned in different GCN modules for topology graph, feature graph and semantic graph are ($Z_{T}$ and $Z_{CT}$), ($Z_{F}$ and $Z_{CF}$), ($Z_{S}$ and $Z_{CS}$), respectively. In order to allow the same type of graph to learn different information in different GCN modules, we adopt the Hilbert-Schmidt Independence Criterion (HSIC) [63] for the embedding learned from the same type of graph, which can enhance the difference of the embedding. For example, the calculation process of constraining $Z_{T}$ and $Z_{CT}$ with the independent criterion is shown in Equation (15) below.

(15) $$L_{T}(Z_{T},Z_{CT})={\left(n-1\right)}^{2}tr(RS_{T}RS_{CT})$$

where $S_{T}$ and $S_{CT}$ are Gram matrices with $S_{T,ij}=S_{T}(Z_{T}^{i},Z_{T}^{j})$ and $S_{CT,ij}=S_{CT}(Z_{CT}^{i},Z_{CT}^{j})$. And $R=I-\frac{ee^{T}}{n}$, where *I* is the identity matrix and e is a column vector. Similarly, $L_{F}(Z_{F},Z_{CF})$ and $L_{S}(Z_{S},Z_{CS})$ can be obtained, and we define the final objective function as $L_{similar}$ is shown in Equation (16) below.

(16) $$L_{similar}=L_{T}(Z_{T},Z_{CT})+L_{F}(Z_{F},Z_{CF})+L_{S}(Z_{S},Z_{CS})$$

Then, we learn three classes of embeddings between different graphs in the common GCN module, namely ($Z_{CT}$ and $Z_{CF}$), ($Z_{CT}$ and $Z_{CS}$), ($Z_{CF}$ and $Z_{CS}$). In order to allow different graphs to learn common information in the same GCN module with shared parameters, these embeddings are calculated using F2-normalization to enhance the dependence between different embeddings, and obtain the loss function $L_{CTF}(Z_{CT},Z_{CF})$. The calculation process of $L_{CTF}$ is shown in Equation (17) below.

(17) $$L_{CTF}=||F_{CT}-F_{CF}|{|}^{2}$$

$F_{CT}$ and $F_{CF}$ are the product of matrices obtained by normalizing the embedding matrices $Z_{CT}$ and $Z_{CF}$ respectively. In the same calculation, $L_{CTS}(Z_{CT},Z_{CS})$ and $L_{CFS}(Z_{CF},Z_{CS})$ can be obtained. We define the final objective function as $L_{common}$, and the calculation process is expressed as shown in Equation (18) below.

(18) $$L_{common}=L_{CTF}(Z_{CT},Z_{CF})+L_{CTS}(Z_{CT},Z_{CS})+L_{CFS}(Z_{CF},Z_{CS})$$

Finally, combined with the constraints of the above three loss functions, we set two hyper-parameters, the disparity constraint coefficient *α* and the consistency constraint coefficient *β*. And define the overall loss function L as shown in equation (19) below.

(19) $$L=L_{cross}+\alpha L_{similar}+\beta L_{common}$$

## 5 Experiments

In this section, we conduct extensive experiments to evaluate the effectiveness of our model for real-world health insurance fraud detection. First, we describe the dataset, baseline models, and related parameter settings used in our experiments. Then we conduct node classification experiments to compare the effectiveness of our model with other baseline models on node classification. In addition, we perform ablation experiments on each of the three types of graphs to verify the effectiveness of the topological structure, feature information, and semantic information extracted by our model. To evaluate the effectiveness of our model on anomaly detection, we perform experiments using the supervised random forest. We also conduct experiments on attention weights to analyze the proportion of different embedded information in the adaptive weight assignment. We modify some parameters to verify the effect of different parameters on the model. Finally, we visualize the nodes of several embedding methods and our model to intuitively analyze the effectiveness of different models for classifying abnormal samples. Overall, these experiments demonstrate the effectiveness of our proposed model for real-world health insurance fraud detection.

### 5.1 Experimental setup

#### 5.1.1 Datasets

We collected two real health insurance datasets from a city's healthcare security administration, and the detailed data information of the health insurance dataset is shown in Table 2. In this study, we treated the same departments in different hospitals in the health insurance dataset as different departments. The Medical-A dataset comprises of comprehensive records of 440 patients, which includes 708 department nodes, 2,328 medicine nodes, and 351 time nodes. There are a total of 3,239 edges connecting patients and departments, 38,956 edges connecting patients and medicines, and 13,348 nodes connecting patients and time. The Medical-B dataset comprises of comprehensive records of over 10,000 patients, which includes 10,647 patient nodes, 2,751 department nodes, 4,718 medicine nodes, and 364 time nodes. There are a total of 48,492 edges connecting patients and departments, 200,407 edges connecting patients and medicines, and 71,442 edges connecting patients and time. The two health insurance datasets, Medical-A and Medical-B, have an equal number of fraudulent samples. Medical-A is a balanced dataset, with a ratio of positive to negative samples being 1:2, while Medical-B is an imbalanced dataset, with a ratio of positive to negative samples being 1:70. We use three meta-paths $P_{PKP}$, $P_{PTP}$, and $P_{PMP}$ in the health insurance data Medical-A and Medical-B. These three meta-paths represent two patients visiting the same department, two patients visiting at the same time, and two patients prescribed with the same prescription, respectively. The meta-graph $P_{PKTMP}$ denotes two patients visiting the same type of hospital department at the same time for the same prescription.

**Table 2:** Statistics of the health insurance datasets.

Datasets	Nodes				Edges			Classes	Features	Positive	Negative
	Patient	Department	Medicine	Time	P-D	P-M	P-T				
Medical-A	440	708	2328	351	3239	38956	13348	2	134	152	288
Medical-B	10647	2751	4718	364	48492	200407	71442	2	134	152	10495

In order to evaluate the effectiveness and generalization ability of our proposed model, we choose two public datasets, ACM and DBLP, for our experiments. Table 3 details the information about the public datasets, including the number of nodes, the number of features, the number of categories, and the number of samples. The ACM dataset has 5,835 papers, 3,025 authors, 56 subjects, and 1,902 terms. The DBLP dataset covers 14,328 papers, 4,057 authors, 20 conferences, and 8,789 terms. In the author categories, four areas are covered, including databases, data mining, machine learning and information retrieval. In the ACM public citation dataset, we sampled three meta-paths and generated meta-graphs. Meta-path $P_{PAP}$ indicates two different articles from the same author; meta-path $P_{PSP}$ indicates two articles belonging to the same discipline; and meta-path $P_{PTP}$ indicates two articles belonging to the same term. The meta-graph $P_{PASTP}$ indicates two articles belonging to the same author, the same type of discipline, and the same term. In the DBLP dataset of academic papers in computer science, we similarly sampled three meta-paths and constructed a meta-graph. The meta-path $P_{APA}$ indicates two co-authors of a paper; the meta-path $P_{APCPA}$ indicates that a paper written by two authors was published in the same conference; and the meta-path $P_{APTPA}$ indicates that a paper written by two authors belongs to the same term. The meta-graph $P_{AP(CT)PA}$ represents two papers by two authors belonging to the same topic and published in the same conference.

**Table 3:** Statistics of the public datasets.

Datasets	Nodes				Features	Classes	Training	Test
ACM	Author(A):5835	Paper(P):3025	Subject(S):56	Term(T):1902	1870	3	60/120/180	1000
DBLP	Author(A):4057	Paper(P):14328	Conference(C):20	Term(T):8789	334	4	80/160/240	1000

#### 5.1.2 Baseline

We compare the MHGSL model with three classes of state-of-the-art algorithms, including generalized graph structure embedding methods, a heterogeneous graph neural network method, and a health insurance fraud detection method. Generic graph embedding methods include GCN, a method for learning node representations by aggregating neighbor information, and GAT [64], a method for aggregating node features using an attention mechanism, there is also MetaPath2vec [65], which is based on the random wandering of meta-paths. Approaches to heterogeneous graph neural networks cover methods based on node-level and semantic-level node embedding HAN [66] as well as network topology and node feature fusion AM-GCN [24]. Approaches to heterogeneous graph health insurance fraud methods include StGNN [67] based on time series and MHAMFD [68] based on multi-layer attention mechanisms.

#### 5.1.3 Parameter setting

During the experiment, we configured several parameters for our models. The learning rate was established at 5e-4, and the weight decay was correspondingly set to the same value. Both the common GCN layer and the specific GCN layer consisted of 2 layers. The first layer had a dimension of 768, and the second layer had a dimension of 256. To avoid overfitting, we incorporated a dropout probability of 0.5 for neural network units. And constrain our model by cross-entropy loss, discrepancy loss and consistency loss. Since the variance of the graph-structured data may be relatively large, to ensure the reliability of the model performance by chance, we repeated each experiment several times during the experiment. The model's performance was also assessed using the corresponding evaluation metrics, a methodology designed to minimize the possible effects of chance caused by the relatively high variance of the graph-structured data. During our experiments, we meticulously established random seeds to ensure reproducibility. For both the initial experiments and subsequent algorithmic comparisons, we employed identical seeds, guaranteeing that each trial or comparison utilized the exact same sequence of random numbers. This rigorous approach ensures the reliability and consistency of our results across different analyses. This initiative is designed to mitigate the impact of stochastic elements on the outcomes of our experiments, thereby enhancing their comparability and reliability. By controlling the random variables, we ensure that the results are a true reflection of the underlying phenomena, rather than artifacts of chance occurrences.

### 5.2 Results and discussion

#### 5.2.1 Node classification

We conducted a classification experiment on a balanced sample of nodes using MHGSL and its three variants (MHGSL-TF, MHGSL-TS and MHGSL-FS) on the Medical-A dataset. To verify the effectiveness of our method, we compared it with four common embedded methods GCN, GAT, MetaPath2vec, HAN. We also used three other methods, AM-GCN, a heterogeneous graph method, and StGNN and MHAMFD, health insurance fraud detection methods, as comparative experiments. To evaluate the effectiveness of the model, we divided the Medical-A dataset into three subsets, namely training, validation, and testing, in different proportions. We assessed the performance of the model using two evaluation metrics, namely accuracy (ACC) and F1 score. We evaluated the effectiveness of sample node classification, and the results are presented in Table 4. Our method slightly underperforms MHAMFD with a small training set and outperforms all other methods in all other cases and achieves better results. We explore the application of the model to a real-world health insurance fraud detection task through binary classification experiments on node classification. We explore how to learn the characteristics of fraud cases from existing samples that have been labeled as anomalous and classify samples with anomalies and samples without anomalies. The difference with the commonly used node classification methods is that we pay more attention to the specificity of the health insurance domain in the data processing and feature extraction stage. By incorporating health insurance domain expertise into the construction of multiple views, we are able to dig deeper into the similarities between different patients, and thus more accurately identify anomalies from a large amount of normal health insurance data.

**Table 4:** Effectiveness of balanced sample node classification.

Train:Val:Test	1:1:3		2:1:2		3:1:1	
Metrics	ACC	F1	ACC	F1	ACC	F1
GCN (Kipf et al., 2016)	0.8674	0.8513	0.7841	0.7535	0.8182	0.7863
GAT (Velickovic et al., 2017)	0.8826	0.8690	0.8750	0.8620	0.8636	0.8601
MetaPath2vec (Dong et al., 2017)	0.7841	0.8056	0.8353	0.8722	0.7680	0.8277
HAN (Wang et al., 2019)	0.8826	0.8729	0.8920	0.8692	0.8636	0.8362
AM-GCN (Wang et al., 2020)	0.8674	0.8558	0.9261	0.9050	0.8864	0.8568
StGNN (Chen et al., 2022)	0.8712	0.8247	0.8750	0.8167	0.8977	0.8615

MHAMFD (Lu et al., 2023)	**0.8961**	0.8694	0.8905	0.8566	0.8757	0.8439
MHGSL-TF (ours)	0.8598	0.8468	0.9318	0.9129	0.8977	0.8727
MHGSL-TS (ours)	0.8636	0.8474	0.8920	0.8630	0.8523	0.8245
MHGSL-FS (ours)	0.8750	0.8634	0.9261	0.9050	0.8864	0.8258
**MHGSL (ours)**	0.8939	**0.8849**	**0.9432**	**0.9284**	**0.8983**	**0.8758**

In order to comprehensively evaluate the effectiveness of MHGSL, we conducted experiments on the node classification task, utilizing two widely used public datasets for graph data mining, ACM and DBLP. For the experiments, we chose three evaluation metrics, namely, Micro-F1, Macro-F1, and Weighted-F1, in order to comprehensively consider the performance of the model in different aspects. The experimental design includes several baseline models, namely GCN, GAT, HAN, and AM-GCN. We chose three different labeling rates, where each category contains 20, 40, and 60 labeled nodes, respectively, in order to evaluate the performance of the models under different labeling distributions more comprehensively. For quantitative evaluation of the model, we randomly selected 1,000 samples from each dataset as a test set to examine the performance of the model in real-life scenarios. The experimental results are shown in Table 5, and in the analysis of the experimental results, we find that MHGSL performs well on both datasets and achieves significant results. This indicates that MHGSL has excellent generalization ability in node classification tasks and can adapt to different domains and data distributions. It proves the superiority of MHGSL in processing complex, multi-domain graph data, and provides strong experimental evidence for research in the field of heterogeneous graph learning.

**Table 5:** Node classification effects on public datasets.

Datasets	Metrics	L/C	GCN	GAT	HAN	AM-GCN	MHGSL
ACM	Micro-F1	20	0.7630	0.8670	0.8540	0.8790	**0.8830**
		40	0.7810	0.8480	0.8470	0.8960	**0.8970**
		60	0.7980	0.8880	0.8870	0.9090	**0.9140**
						
	Macro-F1	20	0.7579	0.8671	0.8534	0.8761	**0.8800**
		40	0.7817	0.8492	0.8478	0.8962	**0.8966**
		60	0.7982	0.8878	0.8863	0.9081	**0.9131**
						
	Weighted-F1	20	0.7572	0.8675	0.8539	0.8768	**0.8807**
		40	0.7815	0.8497	0.8482	0.8966	**0.8970**
		60	0.7981	0.8882	0.8868	0.9085	**0.9136**

DBLP	Micro-F1	20	0.6640	0.7600	0.7240	0.7950	**0.8040**
		40	0.7000	0.7800	0.7660	0.8070	**0.8650**
		60	0.7310	0.7830	0.7610	0.8141	**0.8680**
						
	Macro-F1	20	0.6667	0.7527	0.7177	0.7860	**0.7942**
		40	0.7034	0.7709	0.7606	0.8088	**0.8570**
		60	0.7258	0.7713	0.7481	0.8039	**0.8567**
						
	Weighted-F1	20	0.6665	0.7590	0.7258	0.7942	**0.8042**
		40	0.7041	0.7775	0.7656	0.8052	**0.8629**
		60	0.7315	0.7792	0.7574	0.8113	**0.8654**

#### 5.2.2 Analysis of variants

To validate the effectiveness of the topological structure, feature information, and semantic information in our model MHGSL, we conducted ablation experiments on the Medical-A dataset by designing three variants MHGSL-TF, MHGSL-TS and MHGSL-FS. MHGSL-TF only contains the topological structure and feature information of nodes without any semantic information, while MHGSL-TS only contains the topological structure and semantic information of nodes without any feature information. On the other hand, MHGSL-FS only contains node feature information and semantic information without any node topology. We compared the classification performance of these three variants with the original model MHGSL to evaluate the contribution of topological structure, feature information, and semantic information to the overall performance of the model.

**Figure 7:**
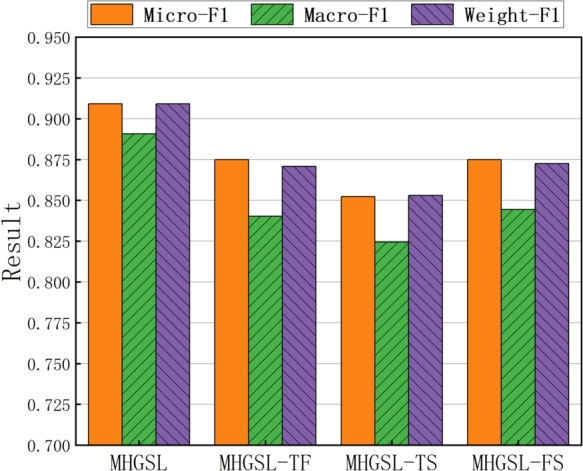
Combined assessment metrics for MHGSL and three model variants in unbalanced data Medical-A.

**Figure 8:**
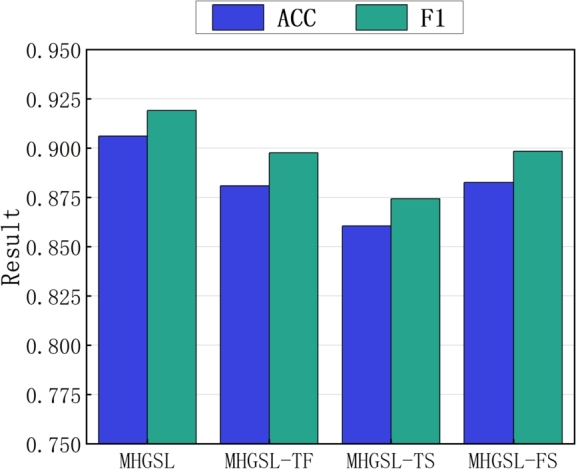
Combined assessment metrics for MHGSL and three model variants in unbalanced data Medical-B.

The classification performance of the three MHGSL variants and the original MHGSL model on different combined evaluation metrics on the balanced health insurance dataset Medical-A is shown in Fig. 7. The results indicate that the classification performance of MHGSL on the three comprehensive evaluation indicators is better than that of the three variant models. Fig. 8 illustrates the classification performance of the three variants and the original model on the unbalanced health insurance dataset Medical-B for different composite evaluation metrics. From the results in the figure, it can be concluded that MHGSL consistently outperforms the other three variants of the model, which illustrates the effectiveness of using feature graphs, topology graphs, and semantic graphs together to obtain feature information. This indicates that our model can fully access the topological, feature, and semantic information of both balanced and unbalanced sample data to extract richer and more useful information for better detection performance.

#### 5.2.3 Fraud detection

In this section, we first train and extract features from health insurance data using a graph neural network-based health insurance fraud detection model. Subsequently, we use the extracted features as inputs and apply the Random Forest algorithm for anomaly detection in downstream tasks. This joint approach aims to improve the accuracy and efficiency of health insurance fraud detection by leveraging the graph neural network model to model the complex relationships of health insurance fraud while combining the advantages of random forest to identify potential anomalous behaviors. Our approach to evaluate the effectiveness of our models in detecting anomalies involves using two real-world health insurance datasets, Medical-A and Medical-B. We aim to validate the performance of various models in anomaly detection tasks using a dataset of balanced and unbalanced samples. To validate the graph neural network models' representation learning capabilities, we obtain a low-dimensional embedding representation and test the performance using a random forest classifier.

The results of the anomaly detection experiments on the balanced sample Medical-A dataset are presented in Table 6. We varied the ratio of the training set, validation set, and test set for the Medical-A dataset during the experiments. To evaluate the anomaly detection performance of the balanced sample nodes, we used the evaluation metrics ACC, Area Under the Curve (AUC), and F1. AUC measures the overall performance of the model in terms of true positive rate and false positive rate, providing a more comprehensive evaluation of the model's effectiveness. As shown in Table 6, our model demonstrates excellent performance in the classification of balanced samples. This can be attributed to the fact that MHGSL is capable of effectively handling multiple relationships between heterogeneous nodes and utilizing the information on relationships, attributes, and similarities between different types of nodes to a greater extent.

**Table 6:** Effectiveness of health insurance fraud detection on the Medical-A dataset.

Train:Val:Test	1:1:3			2:1:2			3:1:1		
Metrics	ACC	AUC	F1	ACC	AUC	F1	ACC	AUC	F1
GCN	0.8106	0.7839	0.7159	0.8920	0.8522	0.8000	0.8295	0.7785	0.6939
GAT	0.8333	0.8215	0.7660	0.8693	0.8639	0.8130	0.8295	0.8125	0.7619
MetaPath2vec	0.7727	0.6747	0.5238	0.8807	0.8081	0.7529	0.8295	0.7339	0.6341
HAN	0.8371	0.8040	0.7456	0.8807	0.8383	0.7789	0.8295	0.7674	0.6809
AM-GCN	0.8598	0.8159	0.7673	0.9034	0.8481	0.8090	0.8636	0.8027	0.7391
StGNN	0.8523	0.8103	0.7636	0.8636	0.8259	0.7857	0.8750	0.8415	0.8070

MHGSL-TF	0.8636	0.8241	0.7778	0.8977	0.8441	0.8000	0.8409	0.8089	0.7308
MHGSL-TS	0.8636	0.8644	0.8125	0.8920	0.8462	0.7957	0.8523	0.7947	0.7234
MHGSL-FS	0.8523	0.8102	0.7578	0.9091	0.8641	0.8261	0.8636	0.8139	0.7500
**MHGSL**	**0.8788**	**0.8517**	**0.8118**	**0.9148**	**0.8681**	**0.8352**	**0.8977**	**0.8604**	**0.8163**

Table 7 presents the results of the anomaly detection experiments conducted on the unbalanced sample Medical-B dataset. To assess the performance of various machine learning models on the Medical-B dataset, we varied the size of the training set to 20%, 40%, and 60% and utilized F1 and Precision as classification evaluation metrics. As we increase the size of the training set, we observed an improvement in the accuracy of the random forest model for the classification of anomalous samples. Additionally, the MHGSL model maintained a consistently high level of effectiveness across all training set sizes.

**Table 7:** Effectiveness of health insurance fraud detection on the Medical-B dataset.

Training Size	Metric	GCN	GAT	MetaPath2vec	HAN	StGNN	**MHGSL**
20%	F1	0.6067	0.7551	0.7103	0.7758	0.8519	**0.8829**
	Precision	0.6296	0.7115	0.7950	0.8041	0.8873	**0.9004**

40%	F1	0.6296	0.7213	0.7166	0.7563	0.8319	**0.8646**
	Precision	0.5894	0.6984	0.8003	0.7981	0.8910	**0.9134**

60%	F1	0.6036	0.7128	0.7321	0.7846	0.8750	**0.8953**
	Precision	0.6121	0.7586	0.8096	0.8116	0.8998	**0.9195**

#### 5.2.4 Classifier performance evaluation

This section evaluates the classification performance of our MHGSL model using different classifiers. Specifically, we focused on three popular classifiers: logistic regression [69], random forest, and Xgboost [70]. By evaluating the performance of the classifiers using these metrics, we can determine which ones are most effective in accurately predicting outcomes for the Medical-B dataset. In this experiment, we will use accuracy, F1 score, and AUC as evaluation metrics to assess the effectiveness of the classifiers on the Medical-B dataset. Evaluating the performance of the classifiers using these metrics, we can determine which ones are most effective in accurately predicting outcomes for the Medical-B dataset.

**Figure 9:**
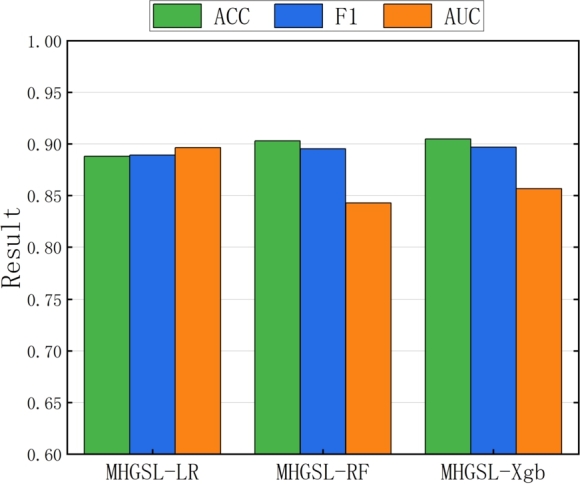
Performance evaluation results of MHGSL on three classifiers.

The classification performance of MHGSL for the three separators is depicted in Fig. 9. The accuracy of random forest and Xgboost is marginally higher than that of logistic regression, which can be attributed to the relatively simplistic nature of logistic regression in comparison to the more intricate techniques employed by random forest and Xgboost. Logistic regression exhibits slightly better classification performance, in terms of AUC, than the other two classifiers, owing to the substantial disparity in the number of positive and negative instances in the Medical-B dataset. The imbalanced distribution of samples can potentially impact the classification efficacy of random forest and Xgboost, whereas logistic regression is more resilient in such scenarios.

#### 5.2.5 Attention analysis

**Figure 10:**
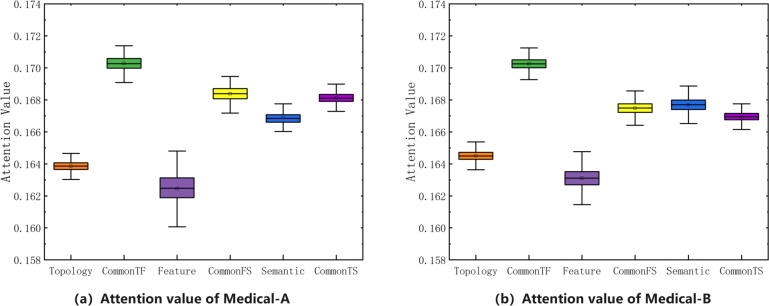
Attention value analysis of different types of embedding assignments.

To analyze the situation where attention assigns weights to different embeddings in the MHGSL model for the Medical-A and Medical-B datasets, we can calculate the proportion of attention weights assigned to each embedding. This analysis can help us understand which embeddings the model is paying more attention to and whether the attention weights are aligned with our expectations based on the importance of the features. Based on the analysis of the proportion of attention weights assigned to different embeddings in the MHGSL model for the Medical-A and Medical-B datasets, we have drawn boxplots of the adaptively assigned attention weight values for different types of embeddings, as shown in Fig. 10. In Fig. 10(a), we can see that the attention weight value of shared parameter space allocation in the Medical-A dataset is higher than that of a single space allocation. This suggests that the model is paying more attention to embeddings learned from the shared parameter space in this dataset. In Fig. 10(b), the average attention weight of the embeddings learned from the shared parameter space of topology and features for the Medical-B dataset has the highest proportion. Moreover, the attention weight of learning semantic embedding assignments in a specific semantic space is higher than that of learning semantic embedding assignments in two common spaces. This suggests that on the Medical-B dataset, the embeddings learned in a single semantic space contain more rich information and are more important for the model's predictions. Overall, these findings provide insights into the attention mechanism of the MHGSL model for the health insurance datasets and can inform further improvements to the model architecture or training process.

#### 5.2.6 Parametric analysis

To investigate the impact of various parameters in the MHGSL model on the classification performance, we conducted experiments on the health insurance dataset Medical-A. We evaluated the model's performance using two common evaluation metrics in multi-class classification tasks Micro-F1 and Macro-F1.

**Figure 11:**
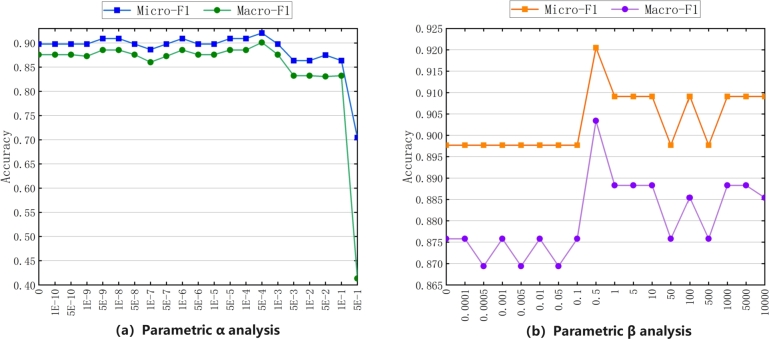
Parameter analysis of consistency constraint coefficient *α* and parallax constraint coefficient *β*.

First, we perform parametric analysis on two hyper-parameters in the loss function: the consistency constraint coefficient *α* and the disparity constraint coefficient *β*. As shown in Fig. 11(a), we set the range of the consistency constraint coefficient *α* from 0 to 0.5. It can be seen that when the value of *α* is in the range of 0 to 0.0001, our model is relatively stable, and the effectiveness of the comprehensive evaluation index F1 is better. However, when the value of *α* increases to 0.001, the validity of the model begins to decline, and after the value increases to 0.1, the validity of the model plummets. As shown in Fig. 11(b), we set the range of the disparity constraint coefficient *β* from 0 to 10,000. The effectiveness of model classification is the best when β=0.5, and the effectiveness of model classification tends to be stable when β≤0.1 and β≥1,000.

**Figure 12:**
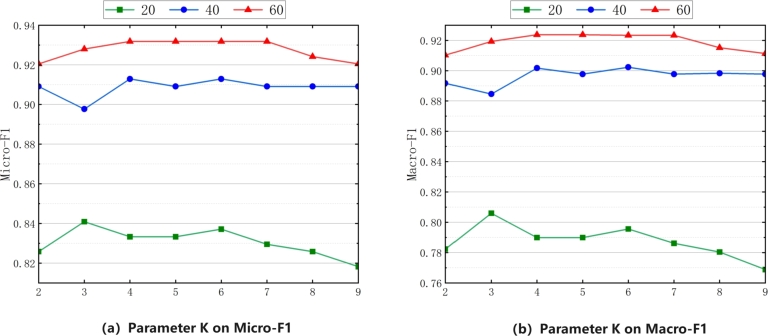
Parameter analysis *k* of the evaluation indexes Micro-F1 and Macro-F1.

We analyze the parameter *k* in the feature graph constructed based on KNN. We set the value of *k* to an integer from 2 to 9 and adjust the epochs to 20, 40 and 60. Fig. 12 (a) analyzes the classification effectiveness of different parameters *k* on Micro-F1, and Fig. 12 (b) analyzes the classification effectiveness of different parameters *k* on Macro-F1. When the number of training rounds is 60 and the parameter *k* is set to 4, the two comprehensive evaluation indicators Micro-F1 and Macro-F1 achieve good classification effectiveness.

#### 5.2.7 Time efficiency analysis

In this section, we compare the efficiency of different channels of MHGSL on the three datasets to explore the effect of different channels on the model time complexity. **w/o Sem.** denotes that it removes the relevant channels of the semantic graph and retains the channels of the topology graph and feature graph. **w/o Fea.** denotes that it removes the relevant channels of the feature graph and retains the channels for the topology graph and semantic graph. **w/o Top.** denotes that it removes the relevant channels of the topology graph and retains the channels for feature graph and semantic graph. **Full.** denotes that it retains the full multi-channel. Table 8 provides a detailed comparison of the training and testing times of the different channels of MHGSL on the Medical-A, ACM, and DBLP datasets. In calculating the efficiency of the training data, we sum and average the epochs of the training rounds. From the table, it is clear that the time overhead of the complete multi-channel is significantly more efficient than removing arbitrary types of channels. And we can find that the time efficiency share of different types of channels is different under different datasets. It is worth noting that on the Medical-A dataset, although the time overhead of the complete multichannel during training is not optimal, there is only about less than one second difference between it and the optimal variant.

**Table 8:** Training and testing time of MHGSL multi-channel in seconds.

Channel	Medical-A		ACM		DBLP	
	train	test	train	test	train	test
w/o Sem.	0.4300	0.1159	1.9446	0.2460	3.2462	0.2567
w/o Fea.	0.6231	0.1950	**1.9125**	0.2328	**2.8722**	**0.1733**
w/o Top.	**0.3378**	**0.1070**	1.9366	**0.2298**	2.9551	0.1943
Full.	0.7114	0.2029	3.1338	0.3325	5.0865	0.2845

#### 5.2.8 Visualization

**Figure 13:**
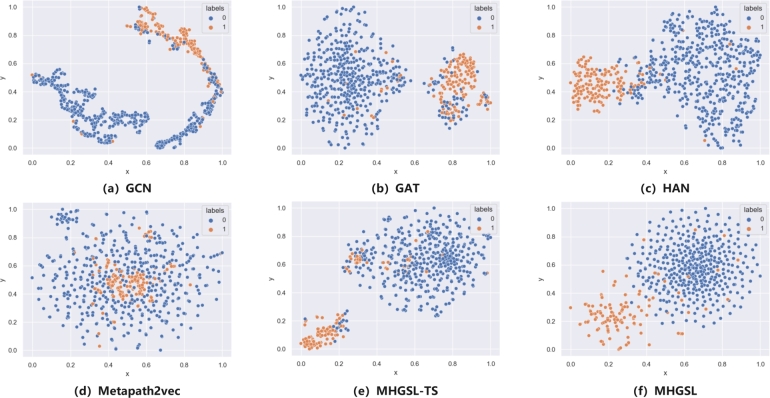
Visualized node embedding learned from health insurance data.

As shown in Fig. 13, our research visualizes the node embeddings learned by different models in the health insurance dataset Medical-B, which makes the classification effectiveness of other common node embedding models in Fig. 13(a-d) and our model in Fig. 13(e-f) more intuitive. And adopt the method of PCA to draw the learned embedding, and visualize the positive and negative samples with different color representations. From the visualized results, we can see that our model has very good properties and an approximately normal distribution.

## 6 Conclusion

In this study, we propose an innovative approach based on multi-channel heterogeneous graph structure learning, named MHGSL, for the health insurance fraud detection problem. Our goal is to develop an algorithm that can effectively identify health insurance fraud and improve fraud detection accuracy by deeply mining the complex structure and intrinsic associations of health insurance data. To achieve this goal, we first extracted the topology of the health insurance data nodes and generated a topology graph. Then, we used the KNN algorithm to aggregate the features of neighboring nodes and create a feature graph. In addition, we sampled and extracted meta-path instances between patients, departments, medicines, and times from the data to obtain high-level semantic information and construct semantic graphs. Finally, we fed these graphs into two specific convolutional networks to adaptively learn node embeddings and weights. Experimental results show that MHGSL performs well on real health insurance datasets and can effectively identify suspicious individuals with a high fraud probability. Compared with some existing embedded methods, our approach has significant advantages in anomaly detection, which proves its potential in the field of health insurance fraud detection. Despite the success of MHGSL, there are some limitations. First, the method relies on high-quality health insurance data, and additional data preprocessing steps may be required for poor data quality. Second, the current model may vary in its ability to identify different types of fraud, and further optimization is needed to improve its generalization ability. In future research, we plan to explore more ways to extract information from health insurance heterogeneous graphs in order to obtain richer and more useful data features. In addition, we will consider introducing knowledge distillation techniques to further improve model performance while preserving and effectively utilizing structure- and feature-based a priori knowledge. We believe that with these improvements, MHGSL will be able to be more effectively applied to health insurance fraud detection and provide stronger support for research and practice in related fields.

## CRediT authorship contribution statement

**Binsheng Hong:** Writing – original draft, Software. **Ping Lu:** Writing – review & editing. **Hang Xu:** Data curation. **Jiangtao Lu:** Validation. **Kaibiao Lin:** Supervision. **Fan Yang:** Visualization, Investigation.

## Declaration of Competing Interest

The authors declare that they have no known competing financial interests or personal relationships that could have appeared to influence the work reported in this paper.

## Data Availability

This study has been approved by the ethics committee/IRB of the China Medical Security Bureau. All methods were performed in accordance with the relevant guidelines and regulations. To ensure the protection of patients' personal information, all datasets underwent anonymization procedures prior to analysis. Permission to access the Medical-A and Medical-B datasets was granted by a city's medical security bureau in China. The informed consent of the experimental subjects was obtained before this study. Due to the confidential nature of the data used in this study, data related to the study could not be shared in a public repository. This decision was made to maintain the confidentiality of the data used in the study in order to comply with relevant regulations and ethical guidelines. These data contain sensitive information, the release of which could lead to privacy breaches and other potentially inappropriate uses. We understand that the public sharing of data is critical to the advancement and transparency of scientific research, but due to the privacy of individuals and the inter-institutional confidentiality agreements involved, we cannot provide public access. The research team is constantly working to balance scientific transparency and data protection within a regulatory and ethical framework.
